# Social Factors and Recovery: A Longitudinal Study of Patients with Psychosis in Mental Health Services

**DOI:** 10.1007/s10597-022-01007-8

**Published:** 2022-08-17

**Authors:** Janniche Linde, Marit Therese Schmid, Torleif Ruud, Regina Skar-Fröding, Eva Biringer

**Affiliations:** 1 Haukeland University Hospital, Bergen, Norway; 2 Western Norway University of Applied Sciences, Bergen, Norway; 3 Division of Mental Health Services, Akershus University Hospital, Lørenskog, Norway; 4 Institute of Clinical Medicine, University of Oslo, Oslo, Norway; 5 Helse Fonna Hospital Trust, Stord, Norway

**Keywords:** Interpersonal relations, Social behavior, Social aspects, Recovery, Psychotic disorder, Mental health services

## Abstract

To study the prospective associations between social factors and recovery in patients with psychotic disorders in mental health specialist services. In this prospective observational cohort study, analyzes were based on baseline- and follow-up data after 18 months from 108 patients with psychosis. Personal recovery was assessed by the Questionnaire about the Process of Recovery (QPR). Linear regression models were used to test the prospective associations between social predictor variables and QPR. An association was found between experienced quality of interpersonal relationships at baseline and change in QPR score over the next 18 months. Stratified analyzes showed that the effect of experienced quality of interpersonal relationships on recovery was due to an association among persons living with others. Patients’ experience of quality of interpersonal relationships are prospectively associated with recovery. In conclusion, findings indicate that interpersonal relationships and social interaction are central drivers of recovery in patients with psychotic disorders.

## Introduction

Relationships with friends and family are important factors for a life outside mental health care. Schön et al. (2009) found that persons who had recovered from severe mental illness described having a close friend who supports and contributes to recovery, this friend may or may not have experience in mental health. Qualitative studies have indicated that people in recovery rarely mention a specific method of treatment as the source of their recovery, but instead tend to refer to a specific person’s presence and actions as helpful (Topor et al., 2006). The recovery process includes not only the personal process in terms of managing mental health challenges, but also how our social life is with focus on friends, work and activity (Topor et al., 2006). In the CHIME framework for personal recovery, connectedness in the form of belonging, meaningful roles and social aspects are central to the personal recovery process (Leamy et al., 2011). However, people with mental health challenges may have fewer close relationships and not all interpersonal relationships may be experienced positively or encouraging for the person and his personal recovery (Tew et al., 2011). Studies on the importance of social relationships and social support for recovery frequently are qualitative studies in the form of in-depth interviews with people who struggle with drug and/or mental health challenges (see for instance Biringer et al., 2017; Greiff et al., 2020). Other qualitative studies reflect the perspective of their professional helpers (Harris & Panozzo, 2019; Roush et al., 2015; Sælør et al., 2015). A recent qualitative study by Hansen et al. (2020), showed that relationships and support from family and friends play an important role in the process of personal recovery. Few studies have investigated the associations between social factors and personal recovery using quantitative methodological approaches. However, Law et al. (2016) in their longitudinal study showed that psychosocial factors were stronger associated with personal recovery than psychiatric symptoms (Law et al., 2016). Hendryx et al. (2009) found that the size of the social network correlated with social support, i.e. the larger the network, the better the social support was experienced by service users. In addition, Hendryx et al. (2009) showed that more involvement in a wide range of activities (physical activity, help groups, activity offers etc.) were also related to recovery. Hendryx et al.s’ (2009) results are consistent with previous studies in the field using quantitative approaches, such as Corrigan and Phelan (2004). These results showed that persons with larger overall network size and more network satisfaction were more likely to report higher scores on the Recovery Assessment Scale (RAS).

Very few longitudinal studies have been performed on the association between social factors and recovery. In a study by Bjornestad et al. (2017), frequency of social interaction with friends was a significant positive predictor of clinical remission over a two-year period. The findings of Bjornestad et al. (2017) imply that increased social frequency, if consistently maintained, may in itself increase subjective satisfaction with social relationships in persons with psychosis. Another study by Bjornestad et al. (2017) showed that frequency of friendship interaction predicted clinical recovery during a two-year period. The authors concluded that positive effects on recovery related to social factors in their study could be attributed to the independent contribution of frequency of interaction with friends. Taken together, several quantitative studies point to an association between social support and recovery in persons with severe mental conditions (Bjornestad et al., 2017; Chronister & Chou 2013; Hendryx et al., 2009; Schön et al., 2009). The studies above provide useful knowledge of the nature and strength of the associations between social relationships and social support in the recovery process. However, there is still need for studies with a long follow-up interval considering multiple social factors as predictors of recovery.

### Aims

The aim of the present study was to investigate the prospective associations between social factors and mental health recovery in a group of patients with psychotic conditions recruited from mental health specialist services. We hypothesized that more and better social contact are associated with recovery over time. We asked the following research questions:

1. Is higher frequency of social contacts with family/relatives or friends prospectively associated with recovery?
2. Is patients’ experienced quality of social relationships prospectively associated with recovery?
3. Is living with others prospectively associated with recovery?
4. Do patients who regard social factors as important for their recovery experience more recovery than patients who do not regard social factors as important?
5. Is experienced support from mental health professionals with regard to social aspects prospectively associated with recovery?

## Methods

### Design

The study had a longitudinal design with data from the project *‘A cluster-randomized study on implementation of guidelines and evidence based treatments of psychoses’* (‘*BedrePsykoseBehandling’).* The present study is a separate substudy based on main trial data. Mental health clinics in six Norwegian health authorities (including three university hospitals) participated in the study. The study was approved by the Regional Committee for Medical and Health Research Ethics (REK Sørøst B 2015/2169), following the principles in the Declaration of Helsinki. Data was gathered in 39 clinical units (i.e. Community Mental Health Centres (CMHCs) and hospital units, including outpatient clinics, day care units, mobile teams and inpatient wards). The inclusion period began in June 2016 and lasted until March 2017. Primary diagnosis of psychotic disorder was inclusion criterion’. The study utilized patient- and therapist-reported data (patient- therapist dyads) to explore the research questions. The study included patient- and therapist reports from inclusion and 18 months follow-up.

### Patients

A total of 325 patient-therapist dyads completed questionnaires at baseline. Due to missing data (N = 86) and patients lost to follow-up (N = 121), the final valid analysis file included responses from N = 108 patient-therapist dyads, i.e., 33% of the patient-therapist dyads at baseline. Among the N = 108 patients in the valid sample, seven were recorded with Mental and behavioral disorders due to psychoactive substance use (F10–F19) as secondary diagnosis, six with Major depressive disorder, single episode or Major depressive disorder, recurrent (F32–F33), eight with Anxiety, dissociative, stress-related, somatoform and other nonpsychotic mental disorders (F40–F48) and four with Disorders of adult personality and behavior (F60–F69). Sixtysix patients (61%) of the 108 patients in the valid analysis file were living alone and 40 (37%) were living with others. Further descriptive statistics are shown in Table 1.

**Table 1:** Sociodemographic and clinical information at inclusion of the sample of patients with valid data at inclusion and 18 months follow-up (N = 108), patients lost to follow-up or excluded (N = 217) and the total sample of patients (N = 325)

Variable	Valid sample (N = 108)		Lost to follow-up or excluded (N = 217)		Total sample (N = 325)	
	Mean (SD)	Ranger	Mean (SD)	Range	Mean (SD)	Range
Age*, years	42 (12.3)	19–77	39 (12.8)	16–71	40 (12.7)	16–77
QPR mean-based total scale	42 (8.62)	17–60	40 (11.1)	5–60	40.9 (10.35)	5–60
GAF-Function	44 (22.8)	10–80	44 (19.7)	10–90	44 (20.7)	10–90
GAF-Symptom	59 (13.4)	30–90	55 (13.2)	30–90	56 (13.3)	30–90

	N	%	N	%	N	%
Sex*						
Male	53	49	138	64	191	59
Female	55	51	78	36	133	41
Missing	0	0	1	0.5	1	0.3
Education, years						
Not completed primary school/junior high school	2	2	7	3	9	3
Completed primary school/junior high school	2	30	64	29	96	30
Completed high school/certificate of apprenticeship	45	42	91	42	136	42
Higher education/college	25	23	38	18	63	19
Other	2	2	8	4	10	3
Missing	2	2	9	4	11	3
Civil status						
Married	10	9	22	10	32	10
Co-habiting/partner	11	10	22	10	33	10
Living alone	74	69	150	69	224	69
Divorced/separated	12	11	21	10	33	10
Widow/-er	1	1	0	0	1	0.3
Missing	0	0	2	1	2	1
Living situation^a^						
Living alone	66	61	138	64	204	63
Living with spouse/partner	18	17	35	16	53	16
Living with children only	6	6	4	2	10	3
Living with parents/family members	13	12	27	12	40	12
Living with friends	4	4	2	1	6	1.8
Missing	1	1	11	5	12	4
Primary diagnosis^b^						
Schizophrenia, schizotypal, delusional, and other non-mood psychotic disorders (F20-F29)	88	81	182	84	270	83
Other diagnosis	11	10	19	9	30	9
Missing	9	8	16	7	25	8
Alcohol or substance* abuse						
Yes	5	4.6	31	14	36	11
No	78	72	147	68	225	69
Missing	25	23	39	18	64	13
Medication						
Yes	104	96	196	90	300	92
No	4	4	13	6	17	5
Missing	0	0	8	4	8	3
Antipsychotic medication^c^						
Yes	93	86	193	89	286	88
No	15	14	24	11	39	12
Missing	0	0	0	0	0	0
Physical disorder						
Yes	47	44	87	40	134	59
No	61	56	130	60	191	41
Work status^d^						
Without work	58	54	107	49	165	51
Ordinary work	10	9	17	8	27	9
Work adjustment	9	8	8	4	17	5
Work training	6	6	24	11	30	9
Student	7	6	16	7	23	7
Stay-at-home parent	1	1	2	1	3	1
Hospitalized	4	4	19	9	23	7
Other	12	11	18	8	30	9
Missing	1	1	6	3	7	2
Professional network^e^						
Community mental health services	50	46	8	4	58	18
Community addiction services	1	1	1	0.5	2	1
Community home care services	15	14	4	2	19	6
Day care/activity centres	33	30.5	3	1	36	11
Private psychiatrist	6	5.5	0	0	6	2
Private psychologist	2	2	0	0	2	1
Missing	1	1	201	93	202	62

*GAF* Global Assessment of Functioning, *QPR* the Questionnaire about the Process of Recovery

*Significant difference between patients with valid data at inclusion and 18 months follow-up (N = 108) versus patients lost to follow-up or excluded (N = 217), Student’s T-tests or Pearson’s chi-square test, *P* < 0.05

^a^When testing the difference between the valid sample of patients (N = 108) versus the patients lost to follow-up or excluded (N = 217), the variable was operationalized as ‘living alone’ (N = 204) versus ‘living with others’ (N = 109)

^b^When testing the difference between between the valid sample of patients (N = 108) versus the patients lost to follow-up or excluded (N = 217), the variable was operationalized as Schizophrenia, schizotypal, delusional, and other non-mood psychotic disorders F20-F29 (N = 270) versus ‘Other disorders’ (N = 30)

^c^Group differences between the valid sample of patients (N = 108) versus the patients lost to follow-up or excluded (N = 217) were only tested for antipsychotic medication

^d^Group differences between the valid sample of patients (N = 108) versus the patients lost to follow-up or excluded (N = 217) were tested for patients currently working (N = 130) versus not working (N = 188)

^e^Patients may use several services and thus numbers do not add up to 325 (100%). No comparisons between groups were performed for this variable

## Measures

### Outcome Variable

Quality of the Process of Recovery (QPR) (Neil et al., 2009) was used as measure of personal recovery. The QPR is a self-completion form with 15 questions for patients with experience of psychosis and their experience of their recovery processes. Examples of items are ‘I feel better about myself’, ‘I feel able to take chances in life’, ‘I feel part of society rather than isolated’ and ‘I feel that my life has a meaning ‘. The 15 items are scored on a five-point scale (0 = Disagree strongly, 1 = Disagree, 2 = Neither agree nor disagree, 3 = Agree, 4 = Agree strongly).

Out of the 325 patients participating at the start of our study, 292 had completed all 15 QPR items. Out of the 121 patients participating in the follow-up at 18 months, 110 had completed all 15 items. Eight patients lacked responses to one item or more, one patient on three items and two patients on four items. Missing items were replaced in up to four of the 15 items in an imputation process similar to ‘bootstrapping’ (Efron & Tibshirani, 1993).

We operationalized the 15 QPR items into a total scale at the start of the study and at 18 months follow-up, respectively. QPR total scales were computed by summing up the 15 item responses (imputed variables) for each patient at each point of measurement. Then, the sum of each of the items was divided by the number of completed items for each patient. Intrascale consistency as measured by Cronbach’s alpha was 0.89 for the QPR total scale in the valid sample at baseline and 0.94 at 18 months follow-up. Finally, a score representing the change in QPR total scale from inclusion to 18 months of follow-up was calculated. The change score was made by subtracting the QPR total score at study start from the QPR total score at 18 months follow-up. A high positive score on the change score means that the patient has experienced more personal recovery from the start of the study to 18 months of follow-up.

### Predictor Variables

Descriptive statistics of social variables, i.e. frequency of social contact, items 4 to 9 of the Interpersonal relationships subscale of the Behavior And Symptom Identification Scale (BASIS-24) and INSPIRE items S1 to S4 at inclusion in the valid sample (N = 108), are shown in Table 2.

**Table 2:** Descriptive statistics of social variables at inclusion in the valid sample (N = 108)

Frequency of social contact	*How often does the patient see the following persons…*				
	Does not have/never	1–4 times/year	1–2 times/month	1–2 times/week	Daily
	N (%)	N (%)	N (%)	N (%)	N (%)
Mother	32 (32)	8 (8)	20 (19)	24 (24)	17 (17)
Father	31 (30)	11 (11)	26 (26)	19 (19)	14 (14)
Children	49 (57)	10 (12)	7 (8)	7 (8)	13 (15)
Brothers or sisters	9 (9)	32 (30)	35 (33)	21 (20)	8 (8)
Relatives	26 (25)	49 (48)	18 (18)	8 (8)	1 (1)
Friends	17 (16)	14 (12)	26 (24)	34 (31)	17 (16)

Interpersonal relationships scale^a^	*During the PAST WEEK, how much of the time did you…*				
	None of the time	A little of the time	Half the time	Most of the time	Always
	N (%)	N (%)	N (%)	N (%)	N (%)
Get along with people in your family (4)	9 (8)	19 (18)	8 (7)	34 (32)	37 (35)
Get along with people outside your family (5)	5 (4)	20 (19)	15 (14)	34 (32)	33 (31)
Get along well in social situations (6)	4 (4)	24 (22)	18 (17)	37 (34)	25 (23)
Feel close to another person (7)	30 (28)	23 (21)	14 (13)	17 (16)	23 (22)
Feel like you had someone to turn to if you needed help (8)	5 (5)	12 (11)	5 (5)	31 (29)	53 (50)
Feel confident in yourself (9)	5 (5)	23 (22)	15 (14)	38 (36)	25 (23)

Whether social factors are important^*b*^	N (%)	Whether one feels supported by the professional helper ^b^				
*An important part of my recovery is…*		*I feel supported by my worker with this…*				
		Not at all	Not much	Somewhat	Quite a lot	Very much
		N (%)	N (%)	N (%)	N (%)	N (%)
Feeling supported by other People (S1)	Yes 95 (88) No 13 (12)	1 (1)	4 (4)	26 (27)	48 (51)	16 (17)
Having positive relationships with other people (S2)	Yes 98 (91) No 10 (9)	1 (1)	11 (11)	21 (21)	50 (51)	15 (15)
Having support from other people who use services (S3)	Yes 67 (64) No 37 (36)	1 (1.5)	3 (4)	28 (42)	28 (42)	7 (10)
Feeling part of my community (S4)	Yes 87 (81) No 21 (19)	0 (0)	14 (16)	23 (26)	39 (45)	11 (13)

^a^Items 4 to 9 of the Behavior And Symptom Identification Scale (BASIS-24)

^b^Items S1 to S4 of the INSPIRE

Due to missing responses, the percentages do not always add up to 100

### Frequency of Social Contact

At baseline, the therapists were asked to answer the following questions in collaboration with their patients: ‘How often does the patient meet the following people?: mother, father, children, siblings, relatives, friends’. Response alternatives for each category of contacts were ‘1’ = ‘Does not have’, ‘2’ = ‘Daily’, ‘3’ = ‘1–2 times/week’, ‘4’ = ‘1–2 times/month’, ‘5’ = ‘I-4 times/year’ or ‘6’ = ‘Never’. We recoded this response scale to a scale from ‘0’ to ‘5’, where ‘0’ = ‘Does not have/Never’, ‘1’ = ‘1–4 times/year’, ‘2’ = ‘1–2 times/month’, ‘3’ = ‘1–2 times/week’ and ‘4’ = ‘daily’. The recoded item score representing frequency of contact with friends was used as predictor variable in the statistical models. We further computed a scale representing frequency of contact with family/relatives by summing up the scores from the items about contact with mother, father, children, siblings and relatives, and then dividing the resulting sum by five (as there were originally five categories). This scale was also used as a predictor variable in the statistical models.

### Patients’ Experience of Interpersonal Relationships

The Behavior And Symptom Identification Scale (BASIS-24) was used to measure patients’ experience of interpersonal relationships. The BASIS-24 is a short self-report questionnaire for patients in mental health developed in the UK (Cameron et al., 2007; Eisen et al., 1999). The questionnaire measures degree of psychopathology and functioning and is filled in by the patient himself (Eisen et al., 1999). According to Cameron et al (2007), BASIS-24 is a brief and easily administered, self-complete measure of mental well-being and functioning that adequately meets the requirements of reliability, validity and responsiveness to change required of an outcome measure. The Behavior and Symptom Identification Scale (BASIS-24) has good validity and reliability for assessing mental health status from a service user perspective (Cameron et al, 2007; Eisen et al, 2004). In this study, we used the six items (items 4–9) which constitute the sub-scale ‘Interpersonal relationships''. These items read as follows: ‘During the PAST WEEK, how much of the time did/have you…’: ‘Get along with people in your family?’, ‘Get along with people outside your family?’, ‘Get along well in social situations?’, ‘Feel close to another person?’, ‘Felt like you had someone to turn to if you needed help?’ and ‘Felt confident in yourself?’. Each item was answered in a 5-point scale from ‘0’ = ‘None of the time’, ‘1’ = ‘A little of the time’, ‘2’ = ‘Half the time’, ‘3’ = ‘Most of the time’ to ‘4’ = ‘Always’. Internal consistency of the subscale as estimated by Cronbach’s alpha was 0.80 among the six items of the Intrapersonal relationships subscale.

### Living Situation

Based on therapist-reported information about the patients’ living situation, respondents were categorized into two sub-groups according to whether the patients lived alone or together with others (Table 1).

### Whether Social Factors were Regarded as Important for Recovery, and Experienced Support with Recovery

We used the INSPIRE Measure of Staff Support for Personal Recovery to examine the patients’ perceptions of the importance of social aspects for their recovery and their experienced support from therapists with their recovery. The INSPIRE is a 27-item self-report questionnaire that measures perceived staff support for personal recovery (Williams et al, 2015). It consists of two subscales: Support (20 items) and Relationships (7 items).

We used three support items from INSPIRE at study start to answer the research questions about whether patients regarded social support as important for their recovery and to what extent they experienced support with their social relationships from their therapists. These items were: ‘Feeling supported by other people’ (item S1), ‘Having positive relationships with other people’ (S2), and ‘Feeling part of my community’ (S4). Patients were asked to answer whether each of the alternatives above were important (‘Yes’) or not (‘No’) to him/her (Part A). They also responded to questions about how much support they received from their therapist in this regard (Part B). Responses were given on a five-point scale: ‘0’ = Not at all’, ‘1’ = ‘Not much’, ‘2’ = ‘Somewhat’, ‘3’ = ‘Quite a lot’ and ‘4’ = Very much’. Means of responses to these three items were used as predictor variables in the statistical models. The item ‘Having support from other people who use services’ was not included as a predictor variable because peer worker support is only available to some patients in specialist mental health services.

### Statistical Analysis

We tested the differences in sociodemographic and clinical variables between the N = 108 sample included in the final valid analysis file and the N = 217 respondents not included with Pearson's chi-square test (categorical variables) or Student's T-tests (continuous variables) (Table 1). Linear regression analyses were used to test the prospective associations between social predictor variables at baseline and change in QPR from baseline to 18 months follow-up. In all models, the QPR total scale at 18 months follow-up was entered as outcome variable and the QPR total scale at baseline as co-variate. Since the QPR total scale at 18-month follow-up was not completely normally distributed, we truncated the lowest variable values ​​into the nearest categories at the outer edges of the distribution curve (histogram). *P*-values were reported from analyses with the truncated QPR-variable as outcome variable. Effect sizes (B and beta) and 95% confidence interval (CI) for B were reported from analyses on the original QPR-variable. The models were adjusted for sociodemographic and clinical variables that may confound the relationship between social factors and recovery. Adjustment variables were age, gender, level of education, level of symptoms (GAF-S), level of functioning (GAF-F), physical conditions (yes/no) and use of medication (yes/no). As the patients’ living situation in terms of whether they lived alone or with others probably moderates an association between social factors and recovery, we choose to stratify the sample with regard to this variable (i.e., living alone versus living with others).We used SPSS v. 24 (Armonk, NY: IBM Corp.). Tests were two-tailed tests with 0.05 as *p*-level.

## Results

Descriptive statistics and statistical comparisons between the final sample (n = 108) and subjects lost to follow-up or excluded due to missing variables (n = 217) are shown in Table 1. Student’s T-tests and Pearson’s chi-square tests showed significant differences for the following variables: age (p = 0.031), sex (p = 0.011) and alcohol-/substance abuse (p = 0.009).

Mean QPR total scale score was 42.0 (standard deviation (SD) = 8.62, range 17–60) at baseline and 40.9 (SD = 10.35), range 5–60, at 18 months follow-up in the valid sample (N = 108). Mean QPR scores at baseline, 18 months follow-up and change from baseline to follow-up are shown in Table 3.

**Table 3:** Descriptive statistics of The Questionnaire about the Process of Recovery (QPR) items- and total scale at inclusion and at 18 months follow-up, and change between inclusion and 18 months, in the valid sample (N = 108)

Variable	Inclusion			18 months follow-up			Change in valid sample from inclusion to 18 months^a^		
	M	SD	Range	M	SD	Range	M	SD	Range
I feel better about myself	3.8	1.07	1–5	3.9	0.99	1–5	0.10	1.07	− 3 to 3
I feel able to take chances in life	3.3	1.06	1–5	3.4	1.11	1–5	0.11	1.23	− 2 to 3
I am able to develop positive relationships with other people	3.9	0.84	2–5	3.8	0.97	1–5	− 0.15	0.97	− 3 to 2
I feel part of society rather than isolated	3.4	1.04	1–5	3.4	1.17	1–5	− 0.04	1.21	− 4 to 3
I am able to assert myself	3.5	0.98	1–5	3.4	1.07	1–5	− 0.16	1.15	− 3 to 3
I feel that my life has a purpose	3.9	0.98	1–5	3.8	1.07	1–5	− 0.13	0.96	− 4 to 2
My experiences have changed me for the better	3.8	0.88	1–5	3.7	1.01	1–5	− 0.15	0.99	− 3 to 3
I have been able to come to terms with things that have happened to me in the past and move on with my life	3.8	0.96	1–5	3.7	1.09	1–5	− 0.09	1.09	− 2 to 3
I am basically strongly motivated to get better	4.1	0.79	2–5	3.9	0.91	1–5	− 0.16	0.94	− 3 to 2
I can recognize the positive things I have done	3.9	0.85	2–5	3.9	0.96	1–5	− 0.08	1.12	− 3 to 3
I am able to understand myself better	3.9	0.81	2–5	3.9	0.88	1–5	− 0.04	1.03	− 3 to 3
I can take charge of my life	3.9	0.83	1–5	3.9	0.92	1–5	− 0.03	0.88	− 2 to 3
I can actively engage with life	3.7	0.98	1–5	3.5	1.06	1–5	− 0.13	0.99	− 2 to 3
I can take control of aspects of my life	3.8	0.86	1–5	3.9	0.89	1–5	0.06	1.05	− 2 to 3
I can find the time to do the things I enjoy	3.9	0.84	2–5	3.8	0.93	1–5	− 0.06	0.90	− 3 to 2

^a^Change variables represent the QPR item score at inclusion subtracted from the QPR item score at 18 months follow-up

### Social Factors and Recovery

Results of the linear regression analyses with social factors at start of the study as predictor variables and change in the QPR total scale as outcome in the total valid sample (N = 108) are shown in Table 4. In the total sample, patients’experienced quality of their social relationships as assessed by the Interpersonal relationships subscale from the BASIS-24, but not their frequency of social contact with friends or family/relatives, was prospectively associated with recovery in terms of increase in QPR total score from start of the study to 18 months follow-up (adjusted B = 2.24 (95% confidence interval for B =  − 0.18; 4.67), *P* = 0.050) (Table 4). No statistically significant associations were found in the analyses with patient’s experience of support from their health care worker, or whether patients regarded social aspects as important for their recovery, as independent variables, and change in QPR from baseline to follow-up as outcome. However, a negative association was found in the models with patients living alone versus living with others as independent variable and change in QPR (adjusted B =  − 3.74 (95% CI − 7.36; − 0.12), p = 0.017). Living alone was associated with increased QPR total score from start of the study to 18 months follow-up.

**Table 4:** Linear regression models with social variables at inclusion as predictor variables and summary scale of The Questionnaire about the Process or Recovery (QPR) at 18 months as outcome variable (N = 108).

Predictor variable		Crude	Adj. demographic variables (age, gender, education)	Adj. demographic variables (age, gender, education) and clinical variables (level of symptoms and functioning, physical conditions and use of medication)
Frequency of contact with friends	B 95% CI for B beta P^b^	1.06 − 0.21; 2.33 0.13 0.080	1.03 − 0.27; 2.34 0.12 0.096	0.81 − 0.56; 2.18 0.10 0.207
Frequency of contact with family/relatives	B 95% CI for B beta P^b^	1.11 − 0.99; 3.20 0.08 0.339	1.04 − 1.21; 3.29 0.08 0.401	0.86 − 1.49; 3.22 0.06 0.532
Interpersonal relationships subscale (BASIS-24, items 5-9^a^)	B 95% CI for B beta P^b^	2.33 0.16; 4.50 0.19 0.020	2.43 0.04; 4.81 0.20 0.032	2.24 − 0.18; 4.67 0.18 0.050
Living with others (yes/no)	B 95% CI for B beta P^b^	− 2.37 − 5.67; 0.93 − 0.11 0.074	− 3.55 − 7.13; 0.034 − 0.16 0.021	− 3.74 − 7.36; − 0.12 − 0.17 0.017
Whether the person regards social aspects as important for their recovery (INSPIRE part A items 1,2,4^a^)	B 95% CI for B beta P^b^	− 1.72 − 8.28; 4.84 − 0.04 0.623	− 2.21 − 8.99;4.57 − 0.05 0.523	− 2.22 − 9.20; 4:76 − 0.05 0.549
Support from health care worker (INSPIRE part B items 1,2,4^a^)	B 95% CI for B beta P^b^	0.62 − 1.68; 2.92 0.04 0.486	0.76 − 1.71; 3.23 0.05 0.440	0.79 − 1.74; 3.31 0.05 0.417

Crude and adjusted analyses

*BASIS-24* 24 item Behavior And Symptom Identification Scale, *CI* confidence interval

QPR summary scale at 18 months was entered as outcome variable and all models were adjusted for QPR summary scale at inclusion

^a^Mean of items entered as independent variable

^b^*P*-values are reported from models with QPR summary scale values truncated at the ends, Bs, 95% CI for B and betas are reported from analyses using nontransformed QPR variables

### Living Alone or Living with Others

The analysis stratified on the basis of whether the patients were living alone (N = 66 (61%)) or with others (N = 41 (37%)) revealed that the significant association between the Interpersonal relationships subscale and change in QPR in the total sample was due to an effect only present in the subsample of patients living with others (adjusted B = 4.89 (95% CI 0.45; 9.33), p = 0.031) (Table 5). In the subsample of patients living alone, there was a trend towards an association between frequency of contact with family/relatives and increase in QPR from start of the study to follow-up (adjusted B = 2.23 (95% CI − 0.37; 4.90), p = 0.102). No such similar trends or associations were found in the subsample living with others.

**Table 5:** Linear regression model with social variables at inclusion as predictor variables and summary scale of The Questionnaire about the Process or Recovery (QPR) at 18 months as outcome variable^a^ (N = 108).

Predictor variable		Living alone (N = 66)		
		Crude	Adj. demographic variables	Fully adjusted model^c^
Frequency of contact with friends	B 95% CI for B beta P	0.85 − 0.75; 2.45 0.10 0.270	0.84 − 0.79; 2.47 0.10 0.306	0.90 − 0.86; 2.66 0.10 0.342
Frequency of contact with family/relatives	B 95% CI for B beta P	2.15 − 0.29; 4.48 0.16 0.093	2.21 − 0.28; 4.70 0.16 0.086	2.23 − 0.37; 4.90 0.17 0.102
Interpersonal relationships subscale (BASIS-24, items 5-9^b^)	B 95% CI for B beta P	1.18 − 1.31; 3.68 1.11 0.207	0.76 − 2.05; 3.57 0.07 0.458	0.79 − 2.14; 3.72 0.07 0.452
Whether the person regards social aspects as important for their recovery (INSPIRE part A items 1,2,4^b^)	B 95% CI for B beta P	− 5.32 − 12.57;1.92 − 0.13 0.168	− 6.14 − 13.43; 1.14 − 0.15 0.110	− 6.00 − 13.81; 1.81 − 0.15 0.154
Support from health care worker (INSPIRE part B items 1,2,4^b^)	B 95% CI for B beta P	0.20 − 2.58; 2.99 0.01 0.834	− 0.02 − 3.02; 2.97 − 0.002 0.938	− 0.23 − 3.43; 2.98 − 0.02 0.880

Predictor variable		Living with others (N = 41)		
		Crude	Adj. demographic variables	Fully adjusted model^e^
Frequency of contact with friends	B 95% CI for B beta P	1.17 − 0.97; 3.31 0.16 0.303	1.22 − 1.03; 3.47 0.17 0.295	1.10 − 1.43; 3.63 0.15 0.846
Frequency of contact with family/relatives	B 95% CI for B beta P	0.62 − 3.46; 4.71 0.05 0.739	− 1.20 − 5.67; 3.27 − 0.09 0.627	− 1.53 − 6.15; 3.10 − 0.11 0.548
Interpersonal relationships subscale (BASIS-24, items 5-9^b^)	B 95% CI for B beta P	6.19 2.17; 10.22 0.45 0.004	5.71 1.36; 10.06 0.41 0.011	4.89 0.45; 9.33 0.35 0.031
Whether the person regards social aspects as important for their recovery (INSPIRE part A items 1,2,4^b^)	B 95% CI for B beta P	6.58 − 6.29; 19.45 0.15 0.325	6.68 − 6.85; 2.21 0.15 0.331	2.83 − 11.33; 16.98 0.07 0.697
Support from health care worker (INSPIRE part B items 1,2,4^b^)	B 95% CI for B beta P	1.39 − 2.71; 5.48 0.11 0.516	2.43 − 2.28; 7.14 0.19 0.297	2.70 − 2.02; 7.42 0.21 0.242

Analyses were stratified based on whether the respondent was living alone or with others. Crude and adjusted analyses

*BASIS-24* 24 item Behavior And Symptom Identification Scale, *CI* confidence interval

QPR summary scale at 18 months was entered as outcome variable and all models were adjusted for QPR summary scale at inclusion

^a^P-values are reported from models with QPR summary scale values truncated at the ends, B, 95% CI for B and beta are reported from analyses using nontransformed QPR variables

^b^Mean of items entered as independent variable

^c^N = 65

^d^N = 40

## Discussion

In the present longitudinal study from specialist mental health services, patients’ experiences of their interpersonal relationships, but not their frequency of social contact, were prospectively associated with personal recovery in terms of increase in QPR from start of the study to 18 months follow-up. Surprisingly, the association of patients’ experiences of their social relationships with recovery was found only in the subsample of patients living with others. In the subsample of patients living alone, there was a trend towards an association between frequency of contact with family/relatives and recovery from start of the study to follow-up. These findings may add to our understanding of the pattern and meaning of social factors in psychosis.

### Prospective Associations Between Frequency of Social Contact and Recovery

To our knowledge, this is one of the first investigations of the prospective associations of social factors with recovery using a quantitative methodological approach and it is the first study with as long follow-up interval as 18 months. The results in this study are consistent with previous quantitative studies showing that social support and social aspects are associated with clinical recovery for patients with mental illness (Bjornestad et al., 2017; Chronister & Chou, 2013; Hendryx et al., 2009). In our study, we found that this was also true for the personal recovery of patients with mental illness. However, although Bjornestad et al. (2017) pointed out that the frequency of social contact with friends is an important positive predictor of recovery, they found no prospective association between frequency of contact with family and recovery (Bjornestad et al., 2017). In the present study, however, there was a trend towards an association between frequency of contact with family/relatives and personal recovery from study start to follow-up in patients living alone. This is consistent with previous findings of associations between social variables and recovery, Hendryx et al. (2009) found that the size of the social network correlated with social support, i.e. the larger the network, the better the social support was experienced by service users. However, our findings should be interpreted with caution, as they are based on small, and potentially biased, subsamples.

### Quality of Social Relationships

We found that experience of social relationships was prospectively associated with recovery. Our results also correspond with the recent qualitative study of Hansen et.al (2020), which showed that relationships and support from family and friends play an important role in the process of recovery. Studies have found that both the size of the persons’ social network and their rating of its supportiveness are associated with their clinical recovery outcomes (Corrigan & Phelan, 2004; Hendryx et al., 2009). Further, it has been shown that the presence of social support from family and friends contribute to better quality of life (Munikanan et al., 2017). For many persons, recovery is a process taking place with support from others within social context (Mezzina et al., 2006). In the CHIME framework, connectedness in the form of belonging, meaningful roles and social aspects are central to the personal recovery process (Leamy et al., 2011). Meeting places, meaningful roles, interplay with others and joyful social activities need to be encouraged and supported by professionals. The association between experienced quality of social contact and recovery in our study supports this notion. Possibly, such support may be particularly important to the subgroup of patients living alone.

### Living with Others or Living Alone

We cannot find current studies exploring the association between living alone or living with others with recovery. One may speculate that frequency of contact or size of the social network may be particularly important for persons living alone. However, in contrast with this notion, our results revealed that the significant association between patients’ experience of their interpersonal relationships and recovery in the total sample was due to an effect only present in the subsample of patients living with others. Again, this finding must be interpreted with caution due to the risk of spurious effects due to attrition or limited statistical power. However, the finding may contradict the notion that social support is particularly important to persons living alone. One alternative explanation for this finding may be that the persons who live with others may be more prosocial in their nature and behavior. Social factors may matter more to persons who are more prosocial and contact seeking. Findings by Waller et al. (2018) show that acknowledgement contributed to the individual's recovery in a myriad of ways. Such acknowledgement may be more helpful for persons who are prosocial and socially active from the beginning on. Our findings also revealed that in the subsample of patients living alone, there was a trend towards an association between frequency of contact with family/relatives and recovery. This may imply that patients who are living alone can seek out family/relatives on their own terms. They also have better opportunities to withdraw from family conflicts or keep their distance whenever they are not feeling their best.

### Strengths and Limitations

The study’ strengths include the fact that it was based on a large sample from 39 specialist mental health services at national level and thus findings may be generalizable to patient groups with psychotic conditions using public mental health specialist services. Further, the study had a longer follow-up interval than previous studies on social factors and recovery. However, the study is hampered by several methodological limitations that need to be mentioned. Firstly, only one third of the baseline sample was available for follow-up after 18 months. This bias may represent a loss of valuable information and may weaken the generalisability of the findings. Comparisons of patients included in the valid analysis file and patients lost to follow-up or excluded, suggest that some biases may exist. Patients included were more often women and older than those excluded or lost to follow-up. Most likely, many of the patients who were lost to follow-up have been referred to primary health care due to improvement in their symptoms and function after baseline measurement. Consequently, the sample studied in the present study most likely represents patients with more severe or chronic problems than the patients who were not followed up. This assumption is supported by the lack of improvement of QPR scores from baseline to follow-up in the total sample. Further, several validated measures were used, but data include only subscales. The construct validity of these subscales with regard to the phenomena we intended to study may be limited, compared to the validity of the complete scales. Furthermore, some social variables were reported by therapists, and some were self-reported by patients. Although both the service user- and the professional perspectives are valid when exploring mental health issues, potential discrepancy between these perspectives in reporting may limit the reliability of the study’s findings. Multiple comparisons were conducted in this study and these may increase the risk of Type 1 error: Finally, there may be factors confounding the associations studied. One limitation is that although the associations studied were adjusted for use of medications, the confounding effect of psychotherapy and psychosocial approaches on the associations studies are not known. However, we argue that several confounding factors likely to influence findings have been adjusted for, as both clinical and sociodemographic factors were included as co-variates in the inferential analyses.

## Conclusions

In the present longitudinal study performed among persons with psychotic conditions using specialist mental health services, an association was found between experienced quality of interpersonal relationships at start of the study and personal recovery over the next 18 months. The finding confirm previous qualitative studies identifying social relationships as central for personal recovery. Further the results of the study indicate that the impact of social factors on recovery may differ between persons living alone versus with others. However, this issue needs further exploration, for instance in investigations on the moderating effects of personal traits on the associations.
