# Correction: Social Factors and Recovery: A Longitudinal Study of Patients with Psychosis in Mental Health Services

**DOI:** 10.1007/s10597-022-01024-7

**Published:** 2022-09-02

**Authors:** Janniche Linde, Marit Therese Schmid, Torleif Ruud, Regina Skar-Fröding, Eva Biringer

**Affiliations:** 1 Haukeland University Hospital, Bergen, Norway; 2 Western Norway University of Applied Sciences, Bergen, Norway; 3 Division of Mental Health Services, Akershus University Hospital, Lørenskog, Norway; 4 Institute of Clinical Medicine, University of Oslo, Oslo, Norway; 5 Helse Fonna Hospital Trust, Stord, Norway

## Correction: Community Mental Health Journal 10.1007/s10597-022-01007-8

The original version of this article unfortunately contained typo in Abstract section.

At the start of the last sentence of abstract, the phrase “In conclusion” needs to be included. Thus, the complete updated abstract will read as follows:

**Abstract**

To study the prospective associations between social factors and recovery in patients with psychotic disorders in mental health specialist services. In this prospective observational cohort study, analyzes were based on baseline- and follow-up data after 18 months from 108 patients with psychosis. Personal recovery was assessed by the Questionnaire about the Pro cess of Recovery (QPR). Linear regression models were used to test the prospective associations between social predictor variables and QPR. An association was found between experienced quality of interpersonal relationships at baseline and change in QPR score over the next 18 months. Stratifed analyzes showed that the efect of experienced quality of interper sonal relationships on recovery was due to an association among persons living with others. Patients’ experience of quality of interpersonal relationships are prospectively associated with recovery. In conclusion, findings indicate that interpersonal relationships and social interaction are central drivers of recovery in patients with psychotic disorders.

The original article has been corrected.

